# Evaluation of Toxicity and Antimicrobial Activity of an Ethanolic Extract from Leaves of *Morus alba* L. (Moraceae)

**DOI:** 10.1155/2015/513978

**Published:** 2015-07-12

**Authors:** Alisson Macário de Oliveira, Matheus da Silva Mesquita, Gabriela Cavalcante da Silva, Edeltrudes de Oliveira Lima, Paloma Lys de Medeiros, Patrícia Maria Guedes Paiva, Ivone Antônia de Souza, Thiago Henrique Napoleão

**Affiliations:** ^1^Departamento de Bioquímica, Centro de Ciências Biológicas, Universidade Federal de Pernambuco, 50670-420 Recife, PE, Brazil; ^2^Laboratório de Farmacologia e Cancerologia Experimental, Departamento de Antibióticos, Universidade Federal de Pernambuco, 50670-420 Recife, PE, Brazil; ^3^Laboratório de Micologia, Departamento de Ciências Farmacêuticas, Centro de Ciências da Saúde, Universidade Federal da Paraíba, 58059-900 João Pessoa, PB, Brazil; ^4^Departamento de Histologia e Embriologia, Centro de Ciências Biológicas, Universidade Federal de Pernambuco, 50670-420 Recife, PE, Brazil

## Abstract

This work evaluated an ethanolic extract from *Morus alba* leaves for toxicity to *Artemia salina*, oral toxicity to mice, and antimicrobial activity. Phytochemical analysis revealed the presence of coumarins, flavonoids, tannins, and triterpenes in the extract, which did not show toxicity to *A. salina* nauplii. No mortality and behavioral alterations were detected for mice treated with the extract (300 and 2000 mg/kg b.w.) for 14 days. However, animals that received the highest dose showed reduced MCV and MCHC as well as increased serum alkaline phosphatase activity. In treatments with the extract at both 300 and 2000 mg/kg, there was a reduction in number of leukocytes, with decrease in percentage of lymphocytes and increase in proportion of segmented cells. Histopathological analysis of organs from mice treated with the extract at 2000 mg/kg revealed turgidity of contorted tubules in kidneys, presence of leukocyte infiltration around the liver centrilobular vein, and high dispersion of the spleen white pulp. The extract showed antimicrobial activity against *Staphylococcus aureus, Pseudomonas aeruginosa, Candida albicans, Candida krusei, Candida tropicalis*, and *Aspergillus flavus*. In conclusion, the extract contains antimicrobial agents and was not lethal for mice when ingested; however, its use requires caution because it promoted biochemical, hematological, and histopathological alterations.

## 1. Introduction

The use of plants as therapeutic tools is based on the popular culture and plant preparations are continually used by people, despite the large number of synthetic drugs developed. This has been mainly associated with the higher costs of allopathic medicines and a limited access of population to them, especially in developing countries where most of the people depend essentially on natural resources to address problems related to diseases of primary care [1]. The relevance of medicinal plants over the centuries has stimulated the search for scientifically proven information on their efficacy and safety for humans [2–4].

*Morus* genus (Moraceae) comprises a group of trees native from Asia, popularly known as mulberries, and with a great importance in folk medicine. In China, the leaves, roots, and branches of* Morus* species are used for treatment of fevers, hepatic protection, vision improvement, strengthening of joints, and reduction of blood pressure [5–7]. The leaves of different mulberries are consumed in Korea and Japan as a nutraceutical food and used for controlling blood glucose levels; this last property is attributed to the presence of the compound 1-deoxynojirimycin, a potent *α*-glucosidase inhibitor [8]. Fukai et al. [9] isolated from* Morus* species a substance deemed chalcomoracin, which showed antimicrobial activity against methicillin-resistant* Staphylococcus aureus*. Dai et al. [10] isolated three compounds from* Morus macroura* bark, called guangsangon H, guangsangon I, and guangsangon J, which showed anti-inflammatory and antioxidant activities.

The pharmacological potential of the species* Morus alba* L. (white mulberry), for benefit of animals and humans, has been investigated. Flavonoids from root showed antiparasitic activity against* Ichthyophthirius multifiliis*, a parasite of gills and skin of freshwater fishes [11] and it was reported that a methanolic extract from the plant foliage might be used as a dietary supplement in order to alleviate* Aeromonas hydrophila* infection in catfish* Clarias gariepinus* [12]. Diels-Alder adducts and prenylated flavanones isolated from the root bark of* M. alba* showed cytotoxic activity against human tumor cells [13]. In another study, a composition containing a blend of standardized extracts from* Uncaria gambir* leaves and* M. alba* root bark was reported to be useful as an alternative therapy for alleviating osteoarthritis and its associated symptoms [14]. Kim et al. [15] reported that extracts from* M. alba* leaves and fruits were able to reduce cognitive deficits in mice induced by the high-fat diet. Other reports mention antioxidant, antibacterial, antiviral, and neuroprotective activities from extracts and isolated compounds from fruits, leaves, stem, and root of* M. alba* [16–20].

In face of the several reports on the medicinal properties of* M. alba* leaves, we investigated in this work the toxicity of an ethanolic extract from this tissue by using two models:* Artemia salina* lethality assay and assessment of* in vivo* oral toxicity to mice. In the last, biochemical, hematological, and histopathological analyses were also performed. In addition, it reports the phytochemical composition of the extract and the evaluation of its antimicrobial activity against pathogens with medical relevance.

## 2. Materials and Methods

### 2.1. Plant Material

Leaves of* M. alba* were collected in Petrolina city, Pernambuco, northeast Brazil. Taxonomic identification was performed and a voucher specimen (number 88372) is deposited in the herbarium of the* Instituto Agronômico de Pernambuco*, Recife, Brazil.

### 2.2. Extract Preparation

The ethanolic extract was chosen for this study because this solvent is able to dissolve both polar and nonpolar substances, including a variety of plant-derived compounds. The leaves were washed with distilled water and dried at 28°C during 24 h and next in an oven at 45°C for 15 days; subsequently, the leaves were powdered. The leaf powder (180 g) was mixed with 70% (v/v) ethanol in distilled water and the mixture was allowed to rest for 48 h, followed by mechanical agitation for 48 h using an orbital shaker. The extract was filtered and the solvent was removed using a rotary evaporator.

### 2.3. Phytochemical Screening

The extract was evaluated for the presence of coumarins, flavonoids, tannins, steroids, alkaloids, anthraquinones, and triterpenes by thin layer chromatography (TLC). The assays were performed using the revealers listed in Table 1 and following the instructions described by Markhan [21], Wagner and Bladt [22], and Abreu [23].

### 2.4. Toxicity to* Artemia salina*

The assay was performed according to Meyer et al. [24] using* A. salina* cysts purchased from San Francisco Bay Brand, Inc. (USA). First, an artificial saline solution (3.8%, w/v) was prepared using sea salt (Marinex, Brazil) and distilled water. In each assay, 10 nauplii were added to extract solutions at different concentrations (100–1,000 *μ*g/mL) prepared by diluting the extract in 10 mL of the artificial saline solution. In controls, the nauplii were incubated with the saline solution. Each concentration was tested in triplicate and three independent experiments were performed. The assays were maintained under artificial lighting for 24 h at 27 ± 2°C. After this period, the mortality rates were determined.

### 2.5. Oral Toxicity Assay

The experiments were performed using female Swiss mice (*Mus musculus*) weighing 38–50 g and reared in the vivarium of the* Laboratório de Farmacologia e Cancerologia Experimental* of the* Universidade Federal de Pernambuco*. The animals are maintained at temperature of 21 ± 1°C, 12L : 12D photoperiod and it is given* ad libitum* access to food (Purina) and water. The experimental procedures were approved by Animal Ethics Committee of the* Universidade Federal de Pernambuco* (process number 23076.061132/2014-33).

The dried extract was dissolved in 0.9% (w/v) NaCl [25] and acute toxicity (mortality and behavioral alterations) was evaluated by oral administration. The mice were separated in three groups (*n* = 3 for each group), according to the instructions of the Organization for Economic Cooperation and Development [26]: control group, which received saline solution (vehicle), and two test groups, which received the extract at 300 mg/kg and 2000 mg/kg b.w. The mice were observed during 14 days.

### 2.6. Biochemical and Hematological Analyses

At the end of oral toxicity assays, the blood of animals was collected and the following biochemical parameters were evaluated: total protein, albumin, alanine aminotransferase (ALT), aspartate aminotransferase (AST), alkaline phosphate, gamma-glutamyl transferase (GGT), urea, and creatinine, using specific kits (Labtest Diagnóstica, Lagoa Santa, Brazil) and a COBAS Mira Plus analyzer (Roche Diagnostics Systems, Basel, Switzerland). Hematologic analysis was performed using an automatic analyzer (Animal Blood Counter: ABC Vet, Montpellier, France) and optical microscopy; the parameters evaluated were as follows: erythrocytes, hemoglobin, hematocrit, mean corpuscular volume (MCV), mean corpuscular hemoglobin (MCH), mean corpuscular hemoglobin concentration (MCHC), and total and differentiated analysis of leukocytes.

### 2.7. Histopathological Analysis

Histological analyses of liver, kidney, and spleen of animals from control and extract treatments were performed by optical microscopy. Fragments of the organs were fixed in buffered formalin (10%, v/v) and then dehydrated through a graded ethanol series (70–100%), diaphonized in xylol, and embedded in paraffin. Histological sections (5 *μ*m) were stained with hematoxylin-eosin and mounted using cover slips with Entellan resin (Merck, Germany) [27]. The materials were observed under a Motic BA200 microscopy coupled to a Moticam 1000 1.3 MP digital camera (Motic Incorporation Ltd., Causeway Bay, Hong Kong).

### 2.8. Antimicrobial Activity

The extract was evaluated for antibacterial activity against three strains of* Staphylococcus aureus* (ATCC-13150, M-177, and LM-197) and two strains of* Pseudomonas aeruginosa* (ATCC-9027 and P-03). Antifungal activity was evaluated against strains of* Candida albicans* (ATCC-76645 and LM-106),* Candida tropicalis* (ATCC-13803 and LM-6),* Candida krusei* (LM-656 and LM-978),* Aspergillus flavus* (LM-118), and* Aspergillus niger* (LM-108). Bacterial suspensions were prepared in Nutrient Broth (Difco Laboratories, France) and fungal suspensions in RPMI 1640 medium (Acumedia, India). The suspensions were standardized for 10^6^ colony-forming units (CFU) per mL using a Leitz-Photometer 340–800 spectrophotometer.

The assays for determination of the minimal inhibitory concentrations (MIC) were performed in U-bottom 96-well microplates. In an assay, each plate well received 100 *μ*L of Nutrient Broth (for bacteria) or RPMI medium (for yeasts and filamentous fungi). Next, 100 *μ*L of the extract, dissolved in distilled water, was added in the wells of the first column of the plate and then a two-fold serial dilution was performed in each row to reach extract concentrations ranging from 32 to 1,024 *μ*g/mL. Finally, 10 *μ*L of microorganism suspension was added to each well. A 100% growth control was done in absence of the extract and positive controls were performed using chloramphenicol (100 *μ*g/mL) for bacteria as well as nystatin (100 IU) and fluconazole (100 *μ*g/mL) for fungi. The plates were incubated for 24 h at 35°C in assays with bacteria and yeasts or for 7–10 days at 28°C in assays with filamentous fungi. After 24 h, bacterial growth was revealed by addition of 20 *μ*L of 0.01% (w/v) resazurin (Inlab Confiança, São Paulo, Brazil); a change in color from blue to red was indicative of microbial growth and thus the MIC was recorded as the lowest extract concentration at which there was no color change [28]. For the fungi, the MIC was defined as the lowest concentration able to inhibit the visual growth in comparison with the 100% growth control. Each antimicrobial assay was performed in duplicate and repeated three times. The antimicrobial activity of the extract was classified according to MIC values as follows: 50–500 *μ*g/mL = strong activity; 600–1500 *μ*g/mL = moderate activity; and >1500 *μ*g/mL = weak activity or inactive [29].

### 2.9. Statistical Analysis

The data were expressed as mean ± SD and submitted to one-way ANOVA followed by Bonferroni's test, with significance level at *p* < 0.05.

## 3. Results and Discussion

Health treatments using medicinal plants are the less expensive and more accessible to the population, mainly in developing countries. However, the indiscriminate use of plant preparations or products with doubtful constitution and without rigorous standardization may pose a risk due to the toxicity of some plant compounds to human organism. For this reason, studies that evaluated the side effects and toxicity degree of preparations and compounds from medicinal plants are imperative. Within this context, this work evaluated the toxicity of an ethanolic extract from leaves of* M. alba*, a plant whose tissues have been extensively used with medicinal purposes, including as commercial formulations.

The phytochemical screening of the extract revealed the presence of coumarins, flavonoids, tannins, and triterpenes (Table 1). Toxicity of the extract was firstly evaluated using the microcrustacean* A. salina*, which is often used as a preliminary indicator of general toxicity of plant compounds; also, it is reported that toxicity to* A. salina* has a correlation with possible antitumor activity [30, 31]. The results showed that the extract had no toxicity at all the tested concentrations, suggesting that it does not have a general toxicity.

In the oral toxicity assays, the groups of mice from control and extract treatments at both 300 and 2000 mg/kg b.w. did not show alterations in behavioral signals during the 14 days of experiments. However, the hematological analysis (Table 2) demonstrated significant (*p* < 0.05) reductions in MCV and MCHC in animals treated with the extract at 2000 mg/kg, in comparison with control; MCHC was also lower than control in treatment at 300 mg/kg. This result indicates that components of the* M. alba* extract may be acting on the erythrocytes causing a reduction of hemoglobin content and may be interfering with the hematopoiesis, causing the production of cells with lower volume. It has been reported that plant secondary metabolites may act directly on erythrocytes interfering with the integrity of plasma membrane and then causing shrinkage, reduction in hemoglobin content, and even destruction of the cells [32].

Table 2 also shows that the number of leukocytes in animals treated with the extract at 2000 mg/kg was significantly lower (*p* < 0.05) than in control animals, having observed a reduction in the proportion of lymphocytes but an increase of segmented leukocytes. At the dose of 300 mg/kg, the extract of* M. alba* also decreased the proportion of lymphocytes and increased the percentage of segmented leukocytes, although the number of total leukocytes was not altered. A possible explanation is that compounds of the extract resulted in a drastic decrease of blood lymphocytes, which stimulated the production of new leukocytes, leading to the increase in proportion of segmented cells. In summary, these results constitute an indication that this extract has an important effect on the immune cells.

Biochemical analysis (Table 3) revealed significant (*p* < 0.05) alterations in the serum levels of the enzymes ALT and alkaline phosphatase in animals that received the extract at 2000 mg/kg, in comparison with control. The group treated with the dose 300 mg/kg did not show significant (*p* > 0.05) variations. Since damage of liver cells usually results in a release of several types of enzymes, we can infer that the* M. alba* extract did not cause strong impairment of hepatocytes membrane. Indeed, no alterations or a decrease only of ALT levels, which is a more specific marker for damage to liver cells, has been interpreted as a signal of stability of the membranes of these cells [33]. An increase only in alkaline phosphatase level is usually indicative of obstruction of bile flow [4] and it is possible that the* M. alba* extract is acting at this level and not by disturbing the membrane of hepatocytes. The levels of GGT, which is a more specific marker for hepatic damage [34], were similar in all groups, corroborating that the* M. alba* extract did not exert hepatotoxic effect.

No differences in food and water consumption were detected between the groups as well as regarding the biomass gain (Table 4). The results from oral toxicity evaluation indicated that the ethanolic extract from leaves of* M. alba* has low toxicity, since no death was observed even in assays at 2000 mg/kg dose [26]. However, the alterations found in hematological and biochemical analyses stimulated us to perform a histopathological study of the livers, kidneys, and spleens of the animals, since damage to these organs may be linked to the changes detected [35].

**Figure 1:**
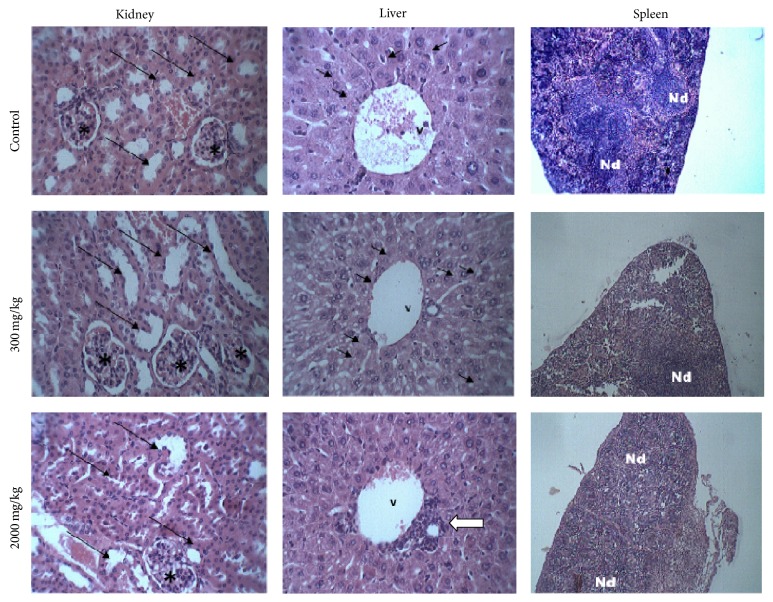
Histopathological analysis of kidneys, livers, and spleens of mice from control and treatments with the ethanolic extract from* Morus alba* leaves at 300 and 2000 mg/kg b.w. Kidneys: in control and treatment with 300 mg/kg, the cortical region with the renal glomeruli (*∗*) and contorted tubules (arrows) without alterations can be seen. In treatment with 2000 mg/kg, a reduction of the subcapsular space and turgid contorted tubules (arrows) is observed. Livers: photomicrography from control treatment shows the centrilobular vein (v) and a preserved organization of the hepatocytes bundles. In the image from treatment with 300 mg/kg, reversible-type cytoplasm vacuolizations (arrows) can be seen while the photomicrograph of an animal that received 2000 mg/kg of extract shows a lymphocyte infiltration around the centrilobular vein (white arrow). Spleens: in control, the well-delimited lymphatic nodes (Nd) can be noticed, while, in images for treatments with 300 and 2000 mg/kg, there is an expansion of lymphatic node (Nd) in the white pulp. All sections were stained with hematoxylin/eosin. Kidney and liver images: 400x. Spleen images: 100x.

Photomicrographs obtained in the histopathological analysis are showed in Figure 1. The structure of the organs was preserved in control animals while a slight presence of reversible-type vacuolization was observed in hepatocytes of animals treated with the extract at 300 mg/kg. On the other hand, all the organs from animals that received the extract at 2000 mg/kg showed alterations. The kidneys showed a decreasing of the subcapsular space and turgidity of the contorted tubules. In the livers, the presence of leukocyte infiltration around the centrilobular vein was detected, which can be associated with intracellular lesions. A high dispersion of the spleen white pulp was also observed, which is a region of lymphatic tissue containing B and T lymphocytes; this can explain the reduction in the number of lymphocytes recorded in hematological analysis.

The ethanolic extract of* M. alba* leaves can be considered more toxic than an ethanolic extract from* Ocimum sanctum* leaves, which did not promote mortality at 200, 600, and 2000 mg/kg in 14 days and did not affect water consumption, body weight, and hematological and biochemical parameters. In addition, there were no alterations in the structure of liver, kidney, and spleen of mice treated with the* O. sanctum* extract at a dose of 800 mg/kg [36]. On the other hand, a leaf extract from* Lawsonia inermis* showed higher toxicity than the* M. alba* extract since it promoted pathological changes in liver and kidney of rats at a dose of 1000 mg/kg, with signals of degenerative/apoptotic changes [37].

Coumarins are known for their hepatotoxicity and have been reported to be toxic to mice and rats, including being able to increase the incidence of adenomas and carcinomas in lungs of rats [38, 39]. Thus, this class of compounds may be involved in the alterations promoted by* M. alba* extract in oral toxicity assays. On the other hand, flavonoids are known to exert hepatoprotective and antioxidant effects [40] and thus may counterbalance the effects of potentially toxic compounds in the extract.

Among the several applications described for* Morus* plants, there is the use for combating infections caused by bacteria and fungi. The increase in the number of microbial strains resistant to the mostly used antibiotics has stimulated the search for new antimicrobial agents, especially those with natural origin [41]. In this way, we evaluated the antimicrobial activity of the ethanolic extract from* M. alba* leaves against bacteria and fungi with medical relevance. The results are summarized in Table 5 and show that the extract presented a strong activity (MIC of 256 *μ*g/mL) against* C. albicans* LM-106 and moderate antimicrobial activity on the other strains, except* A. niger*, which was not sensitive to the extract. The bacterium most sensitive to the extract was* S. aureus* ATCC-13150. The strains* C. albicans* ATCC-76645,* C. albicans* LM-106, and* C. krusei* LM-656 were resistant to the positive controls used.

## 4. Conclusions

Ethanolic extract of* M. alba* leaves showed low oral toxicity to mice since no animal death was detected at a dose of 2000 mg/kg. However, the extract promoted biochemical, hematological, and histopathological alterations at this dose, which indicates that caution is required regarding its use. At 300 mg/kg, the extract did not show toxicity or cause irreversible cellular damages but altered the proportion of leukocyte types. The extract showed mainly moderate activity against the microbial pathogens evaluated.

## Figures and Tables

**Table 1:** Phytochemical screening of ethanolic extract from *Morus alba *leaves through thin layer chromatography.

Compound classes	Revealer	Result
Alkaloids	Dragendorff's reagent	Negative
Anthraquinones	KOH	Negative
Coumarins	KOH	Positive
Steroids	Liebermann-Burchard	Negative
Flavonoids	Aluminum chloride	Positive
Tannins	Iron (III) chloride	Positive for condensed tannins
Triterpenes	Liebermann-Burchard	Positive

**Table 2:** Hematological parameters of animals from control group and treated with ethanolic extract of *Morus alba* leaves for 14 days.

Parameter	Control	Extract	
		300 mg/kg b.w.	2000 mg/kg b.w.
Erythrocytes (10^6^/mm^3^)	7.05 ± 0.39	7.84 ± 0.58	6.82 ± 0.51
Hematocrit (%)	39.95 ± 0.21	41.47 ± 2.41	36.50 ± 4.95
Hemoglobin (g/dL)	17.35 ± 1.20	15.62 ± 1.05	16.00 ± 1.41
MCV (fL)	64.00 ± 5.00	64.33 ± 5.03^Δ^	50.00 ± 1.00^*∗*Δ^
MCH (pg)	26.97 ± 1.91	24.80 ± 0.85	21.20 ± 4.53
MCHC (%)	38.20 ± 1.01	32.67 ± 0.85^*∗*^	32.20 ± 2.01^*∗*^
Leukocytes (10^3^/mm^3^)	8.26 ± 0.43	9.57 ± 0.66^Δ^	6.81 ± 0.27^*∗*Δ^
Segmented (%)	22.68 ± 1.16	43.90 ± 3.41^*∗*Δ^	55.71 ± 3.12^*∗*Δ^
Lymphocytes (%)	58.88 ± 1.70	42.39 ± 2.57^*∗*Δ^	29.65 ± 0.92^*∗*Δ^
Monocytes (%)	18.44 ± 3.55	13.71 ± 1.35	14.61 ± 0.64

^*∗*^Significantly different (*p* < 0.05) from control treatment. ^Δ^Significantly different (*p* < 0.05) from the other dose of *M. alba *leaf extract. MCV: mean corpuscular volume; MCH: mean corpuscular hemoglobin; MCHC: mean corpuscular hemoglobin concentration.

**Table 3:** Biochemical parameters of blood of animals from control and treatments with the ethanolic extract from *Morus alba *leaves for 14 days.

Parameters	Control	Extract	
		300 mg/kg b.w.	2000 mg/kg b.w.
Albumin (g/dL)	2.14 ± 0.17	1.92 ± 0.13	2.25 ± 0.25
ALT (U/L)	78.50 ± 2.12	80.3 ± 8.02^Δ^	66.33 ± 4.73^*∗*Δ^
AST (U/L)	147.50 ± 4.95	149.67 ± 6.81	149.33 ± 5.03
Total protein (g/dL)	6.16 ± 0.23	6.09 ± 0.35	6.13 ± 0.17
Alkaline phosphatase (IU/L)	14.00 ± 1.0	12.33 ± 2.52^Δ^	37.00 ± 2.65^*∗*Δ^
GGT (U/L)	11.00 ± 1.41	12.67 ± 1.15	14.00 ± 1.50
Urea (mg/dL)	54.33 ± 4.93	50.33 ± 1.53	53.33 ± 2.08
Creatinine (mg/dL)	0.28 ± 0.02	0.28 ± 0.02	0.30 ± 0.04

^*∗*^Significantly different (*p* < 0.05) from control treatment. ^Δ^Significantly different (*p* < 0.05) from the other dose of *M. alba *leaf extract.

**Table 4:** Evaluation of food and water consumption and weight gain of animals from control and treated with the ethanolic extract from leaves of *Morus alba*.

Parameter	Control	Extract	
		300 mg/kg b.w.	2000 mg/kg b.w.
Water consumed (mL)	35.00 ± 1.05	34.82 ± 1.68	34.29 ± 1.27
Food consumed (g)	28.68 ± 1.23	28.13 ± 1.00	27.09 ± 1.28
Weight gain (g)	12.04 ± 0.79	11.98 ± 0.49	12.00 ± 0.38

**Table 5:** Minimal inhibitory concentrations (MIC) of ethanolic extract from *Morus alba *leaves against bacteria and fungi.

Microbial strain	MIC (*µ*g/mL)
*Staphylococcus aureus *ATCC-13150	512
*Staphylococcus aureus *M-177	1,024
*Pseudomonas aeruginosa *ATCC-9027	1,024
*Pseudomonas aeruginosa *ATCC-P-03	1,024
*Candida albicans *ATCC-76645	1,024
*Candida albicans *LM-106	256
*Candida tropicalis *ATCC-13083	512
*Candida tropicalis *LM-6	1,024
*Candida krusei *LM-656	512
*Candida krusei *LM-978	512
*Aspergillus flavus *KM-714	512
*Aspergillus niger *LM-108	NI

NI: no inhibition.
